# The Center for Epidemiologic Studies Depression Scale: A Review with a Theoretical and Empirical Examination of Item Content and Factor Structure

**DOI:** 10.1371/journal.pone.0058067

**Published:** 2013-03-01

**Authors:** R. Nicholas Carleton, Michel A. Thibodeau, Michelle J. N. Teale, Patrick G. Welch, Murray P. Abrams, Thomas Robinson, Gordon J. G. Asmundson

**Affiliations:** 1 The Anxiety and Illness Behaviour Laboratory, Department of Psychology, University of Regina, Regina, Saskatchewan, Canada; 2 Regina Qu’Appelle Health Region, Functional Rehabilitation Program, Regina, Saskatchewan, Canada; Tehran University of Medical Sciences, Iran (Republic of Islamic)

## Abstract

**Background:**

The Center for Epidemiologic Studies Depression Scale (CES-D; Radloff, 1977) is a commonly used freely available self-report measure of depressive symptoms. Despite its popularity, several recent investigations have called into question the robustness and suitability of the commonly used 4-factor 20-item CES-D model. The goal of the current study was to address these concerns by confirming the factorial validity of the CES-D.

**Methods and Findings:**

Differential item functioning estimates were used to examine sex biases in item responses, and confirmatory factor analyses were used to assess prior CES-D factor structures and new models heeding current theoretical and empirical considerations. Data used for the analyses included undergraduate (n = 948; 74% women), community (n = 254; 71% women), rehabilitation (n = 522; 53% women), clinical (n = 84; 77% women), and National Health and Nutrition Examination Survey (NHANES; n = 2814; 56% women) samples. Differential item functioning identified an item as inflating CES-D scores in women. Comprehensive comparison of the several models supported a novel, psychometrically robust, and unbiased 3-factor 14-item solution, with factors (i.e., negative affect, anhedonia, and somatic symptoms) that are more in line with current diagnostic criteria for depression.

**Conclusions:**

Researchers and practitioners may benefit from using the novel factor structure of the CES-D and from being cautious in interpreting results from the originally proposed scale. Comprehensive results, implications, and future research directions are discussed.

## Introduction

The Diagnostic and Statistical Manual of Mental Disorders, Fourth Edition, Text Revision [1] characterizes depression as a multidimensional construct comprising negative emotion (i.e., negative affect; Criterion A1), an absence of positive emotions (i.e., anhedonia; Criterion A2), and a cluster of physical symptoms (i.e., somatisation; Criteria A3-5). The Center for Epidemiologic Studies Depression Scale (CES-D) [2] is among the most popular measures of depressive symptoms, likely owing its popularity to being free and generally comparable [3]–[5] with the well-established Beck Depression Inventories [6], [7]. Despite its popularity, the CES-D has areas of concern, particularly in its latent factor structure and item content.

The CES-D was originally posited as having a 4-factor structure representing depressed affect, absence of positive affect or anhedonia, somatic activity or inactivity, and interpersonal challenges [2]. The CES-D items and structure were not designed a priori to reflect diagnostic criteria at the time of its development [8] and recent investigations have called into question the robustness and stability of the original 4-factor 20-item structure [9]–[11]. Indeed, over 20 alternative factor solutions have been reported (Table 1) and have suggested the presence of one, two, three, and four factors [12]–[14]. The majority of factor-analytic studies of the CES-D have employed principal component analysis with orthogonal rotation [4], an analytic approach that may have theoretically improbable assumptions and biased factor solutions [15]. The shift away from such approaches is not a shift away from exploratory factor analyses, but a shift towards the best practices for such analyses; that said, exploratory factor analyses tend to be exploratory. In the case of constructs that are established (e.g., depression), confirmatory factor analyses may be more informative as measures are designed to fit a construct, instead of naming constructs to fit the results from a measure.

**Table 1:** Prior multi-factorial model structures sorted by publication date.

	Factors (Items)	CES-D Item Number and Posited Factor Loading																			
Reference		1	2	3	4	5	6	7	8	9	10	11	12	13	14	15	16	17	18	19	20
Radloff, 1977 Model A [2]	4 (16)	1	1	2	4		2	1	4			1	4		2	3	4	2	2	3	1
Radloff, 1977 Model B [2]	4 (20)	1	1	2	4	1	2	1	4	3	3	1	4	1	2	3	4	2	2	3	1
Radloff, 1977 Model C [2]; Shafer, 2006 [4]; Williams, 2007 [14]	4 (20)	1	1	2	4	1	2	1	4	2	2	1	4	1	2	3	4	2	2	3	1
Burnam, 1988 [66]; Tuunainen, 2001[67]	1 (6)						1					1					1	1	1	1	
Shrout, 1989 Model A [58]	1 (5)						1					1			1			1			1
Shrout, 1989 Model B [58]	1 (5)						1		1				1					1		1	
Radloff, 1991 [47]	4 (17)	1	1	2	4	1	2	1	4			1	4		2	3	4	2	2	3	1
Kohout, 1993 [21]; Carpenter, 1998 [59]; Irwin, 1999 Model A [60]	4 (19)	1	1	2	4	1	2	1	4	4		1	4	1	2	3	4	2	2	3	1
Kohout, 1993 [21]; Carpenter, 1998 [59]; Irwin, 1999 Model B [60]	4 (11)		1				2	1				1	4		2	3	4		2	3	1
Kohout, 1993 [21]; Carpenter,1998 [59]; Irwin, 1999 Model C [60]	4 (10)						1	2				2	4		1	3	4		1	3	2
Andresen, 1994 [40]; Cheng, 2006 [16]	3 (10)	1				2	1	2	3		1	2	3		1						2
Santor, 1997[61]; Herrero, 2006 [62]	1 (9)	1		1		1	1	1				1	1				1		1		
Boey, 1999 Model A [63]	1 (10)	1				1	1	1	1		1	1	1		1						1
Boey, 1999 Model B [63]	2 (10)	1				1	1	1	2		1	1	2		1						1
Rouch-Leroyer, 2000 [64]	1 (5)						1		1				1						1		1
Schroevers, 2000 [10]; Rivera-Medina, 2010 [29]	2 (20)	1	1	1	2	1	1	1	2	1	1	1	2	1	1	1	2	1	1	1	1
Yen, 2000 [65]	3 (17)		1	1	4	1	1	1	4			1	4	2	2	2	4	2	2	2	1
Bush, 2004 Both Sexes [68]	4 (20)	1	3	1	4	1	1	1	4	2	1	1	4	3	2	2	4	3	2	2	1
Bush, 2004 Men Only [68]	3 (20)	1	1	2	3	1	1	1	3	2	1	1	3	2	2	2	3	2	2	2	1
Bush, 2004 Women Only [68]	4 (20)	1	3	1	4	1	1	3	4	1	1	3	4	3	2	2	4	1	2	2	3
Cole, 2004 [69]	4 (10)	1		2	3	1		1	3	4	4				2	4					
Stansbury, 2006 [11]	3 (16)	1	1	2		1	2	1		3	2	1		1	2	3		2	2	3	1
Lee AE, 2008 [22]	2 (10)	1		1		1	1		2		1	1	2		1						1
Lee SW, 2008 Model A [13]	2 (16)	1	1	1		1	1	1		1	2	1		2	2	2		2	2	2	1
Lee SW, 2008 Model B [13]	3 (16)	1	1	2		1	2	1		3	3	1		1	2	3		2	2	3	1

Notes: For clarity, the first author’s name and the year are provided to help identify models; CES-D = Center for Epidemiological Studies Depression scale.

Many researchers have also questioned the validity and psychometric properties of several items on the CES-D [16]–[25]. Items potentially assessing somatic concerns (e.g., “I felt that everything I did was an effort”) may artificially inflate CES-D scores for elderly or chronic pain populations [26], [27]. Two socially-focused items (i.e., “People were unfriendly” and “I felt that people disliked me”) are believed to potentially confound the validity of the CES-D by assessing other constructs (e.g., perceived social competence) and symptoms of other disorders (e.g., Social Anxiety Disorder) [4], [11], [14], [21]. For at least one item (i.e., “I had crying spells”), there appears to be a robust sex difference in responses, leading to inappropriate inflation of women’s CES-D scores due to cultural norms regarding emotional expression, rather than actual differences in depressive symptoms [19], [20], [25], [28], [29]. Furthermore, the CES-D also includes four reverse-worded items (e.g., “I was happy”) designed “…to break tendencies toward response set as well as to assess positive affect (or its absence)” [2]; however, these two purposes are at odds and may lead to misrepresentation of response patterns or biased estimations of positive affect [4], [30]. Research suggests that depression marked by absence of positive affect (i.e., anhedonia) may be qualitatively and quantitatively different than depression resulting from heightened negative affect [31]–[33], implying that measures of depression should assess this dimension directly.

The aims of the current study were to (1) identify any sex biases within the item content of the CES-D, (2) explore which of the many prior factor solutions for the CES-D (Table 1) would demonstrate the best factorial validity, and (3) test whether a new theory-driven solution would exhibit the best fit. The ability of items to predict depression similarly among men and women (i.e., differential validity) was assessed by using an application of item response theory. The factorial validity of the CES-D was examined using a series of confirmatory factor analyses (CFAs) that tested previously established models, as well as new models based on theory and empirical research. This approach is in line with conclusions from a recent meta-analysis [4] suggesting that the use of CFAs would be an appropriate next step in solidifying the optimal factor structure of the CES-D; that is, the use of CFAs will circumvent the almost exclusive prior use of exploratory factor analytic techniques with the CES-D [4], [15]. The present study performed these analyses using five different samples (i.e., undergraduate, community, rehabilitation, clinical with a history of depression, and a nationally representative sample from the National Health and Nutrition Examination Survey; NHANES) to permit generalizability of the findings across several applications (e.g., epidemiological, clinical), while addressing the overuse of data from specialized samples in this area (e.g., adolescent, geriatric).

## Methods

### Ethics Statement

The present study has been ethically approved by the University of Regina Research Ethics Board. The study uses archival data from several sources (details below); however, participants provided written informed consent prior to participating in the data collection associated with each archival source. The consent forms in those data collections were all approved by ethics committees.

### Participants

The first sample included undergraduates (n = 948) from the University of Regina (251 men, 18–52 years [M age = 21.2; SD = 4.3] and 697 women, 18–50 years [M age = 21.0; SD = 4.7]) who completed the CES-D as part of other investigations approved by the University of Regina Research Ethics Board. Using this type of sample generally ensures a wide range of responses, whereas an entirely clinical sample might provide a restricted range of relatively higher responses [15], [34]. Participants identified their ethnicity as White/Caucasian (89%), First Nations (i.e., Canadian Aboriginal; 3%), Asian (4%), or other (4%). Most reported being single (84%), while others were married or cohabiting (13%), separated or divorced (1%), or chose not to answer (2%). Undergraduates were recruited via campus advertisements directing them to a secure website for completion of an online questionnaire package.

The second sample included community members (n = 254) from across Canada (73 men, 18–54 years [M age = 32.6; SD = 11.3] and 181 women, 18–55 years [M age = 32.0; SD = 11.3]) who completed the CES-D as part of another web-based investigation approved by the University of Regina Research Ethics Board. Like the undergraduate sample, the community sample was included to ensure a wide range of responses. Most (70%) reported having at least some postsecondary education, being employed (50% full-time, 14% part-time, 10% as homemakers), and being single (52%). Others reported being married or cohabiting (35%), separated or divorced (10%), or chose not to answer (3%). Participants identified their ethnicity as Caucasian (87%), First Nations (Canadian Aboriginal; 2%), Asian (2%), or other (9%).

The third sample was a rehabilitation sample of tertiary level rehabilitation patients (n = 522) from a government-sponsored rehabilitation program who completed the CES-D as part of tertiary assessment for issues related to injuries sustained in motor-vehicle or work place accidents (246 men, 18–85 years [M age = 42.5; SD = 12.5] and 276 women, 18–79 years [M age = 43.2; SD = 12.5]). The rehabilitation sample was included to provide a comparatively broad range of responses from a treatment-seeking sample that is very likely distressed, but not necessarily depressed. Ethnicity data was not recorded for the rehabilitation sample, but can be assumed to be primarily Caucasian based on population demographics. Most reported being married or cohabiting (57%), while others were single (27%), separated or divorced (13%), or widowed (3%). Education levels were not available for this sample.

The fourth sample, described as a clinical sample, included community members (n = 84) from across Canada (19 men, 18–53 years [M age = 29.4; SD = 11.4] and 65 women, 18–55 years [M age = 24.4; SD = 8.4]) who completed the CES-D as part of another web-based investigation approved by the University of Regina Research Ethics Board. In this sample, participants reported being diagnosed with Major Depressive Disorder by a psychiatrist (77%) or a registered doctoral level psychologist (23%). The average reported length of time since diagnosis was approximately four years. Most of the clinical participants (60%) reported having at least some postsecondary education, and most reported being employed (24% full-time, 20% part-time) or students (39%). Clinical participants identified their ethnicity as Caucasian (89%), First Nations (i.e., Canadian Aboriginal; 4%), or other (7%). Participants reported being single (63%), married or cohabiting (29%), or separated or divorced (8%).

The fifth sample, referred to throughout as the NHANES sample, included community members (n = 2814) from a large scale sampling of participants across the United States (1242 men, 25–74 years [M age = 46.5; SD = 14.0] and 1572 women, 25–74 years [M age = 45.1; SD = 13.9]) who completed the CES-D. The data was collected by the National Center for Health Statistics from 1971–1975 as part of a Health and Nutrition Examination Survey; however, depression symptoms have not changed substantially since then [1], [8]. The public access data is from the National Institute of Mental Health and we are grateful for the NHANES contribution. Comprehensive descriptions of the data collection are available directly online from the Centres for Disease Control and Prevention. Many of the NHANES participants reported having completed Grade 12 (37%) or having at least some postsecondary education (32%), and most reported being employed (52% full-time, 11% part-time) or working as homemakers (33%). NHANES participants identified their ethnicity as Caucasian (91%), African American (8%), or other (1%). The majority reported being married or cohabiting (79%), while others reported being single (7%), separated or divorced (8%), or widowed (6%).

### Measures

The CES-D is a 20-item measure assessing symptoms of depression with items phrased as self-statements (e.g., “I felt hopeful about the future”). Respondents rate how frequently each item applied to them over the course of the past week. Ratings were based on a 4-point Likert scale ranging from 0 (rarely or none of the time [less than 1 day]) to 3 (most or all of the time [5–7 days]).

### Analyses

#### Descriptive statistics and differential item functioning

Descriptive statistics were calculated for each item within each of the samples (Table 2). Means on each of the items for men and women were compared by t-tests across samples as an initial index of differential validity. Differential item functioning was subsequently estimated to assess whether men and women differed in their responses to each item along the continuum of CES-D scores. Differential item functioning occurs when individuals with the same latent trait (i.e., depression) or total score (e.g., on the CES-D) respond to items differently due to test characteristics (e.g., paper and pencil vs. computerised) or biases (e.g., due to sex or race [35], [36]). Estimates of differential item functioning can illustrate, for example, that men and women may respond similarly to an item when they have relatively low CES-D scores, but respond differently to the item when they are severely depressed. Differential item functioning was estimated using an item response theory approach rather than a Mantel-Haenszel approach as it provides a more accurate estimate of non-uniform differential item functioning (e.g., if it occurs only in more severe levels of depression [37]). Non-parametric item characteristic curves were rendered using jMetrik 2.1.0 [38] and were smoothed using a Gaussian kernel. Item characteristic curves are an integral part of item response theory that plot which response option (e.g., 0, 1, 2, or 3 on a Likert scale) is most likely to be endorsed by an individual with a certain total score. To illustrate an absence of differential item functioning on the CES-D, men and women with similar levels of depression should endorse the same option on each item of the CES-D (e.g., severely depressed men and women would both chose the highest option), and therefore exhibit very similar item characteristic curves. The distance between the curves for each sex was examined manually to identify potential differential item functioning. An item was only confidently deemed to exhibit differential item functioning if the curves for men and women were grossly dissimilar either in slope or intercept. Item response theory analyses require both relatively large samples and a range of scores spanning the full continuum of potential scores on the measure [35]; consequently, all five samples were combined for these, but not subsequent analyses. Item characteristic curves were plotted based on total CES-D scores, rather than latent depression, given the aforementioned difficulties associated with the latent structure of the CES-D.

**Table 2:** Descriptive statistics.

	Undergraduate Sample (n = 948)			Community Sample (n = 254)			Rehabilitation Sample (n = 522)			Clinical Sample (n = 84)			NHANES (n = 2814)		
	M (SD)	S (.08)	K (.16)	M(SD)	S (.15)	K (.30)	M (SD)	S (.11)	K (.21)	M(SD)	S (.26)	K (.52)	M (SD)	S (.05)	K (.09)
CES-D 1	.58 (.79)	1.26	.96	.54 (.76)	1.20	.57	.87 (.94)	.77	−.44	1.27 (.97)	.14	−1.02	.34 (.66)	2.11	4.22
CES-D 2	.58 (.85)	1.40	1.15	.75 (.96)	1.02	−.14	.82 (1.00)	.90	−.43	1.61 (1.16)	−.17	−1.43	.29 (.69)	2.56	5.98
CES-D 3	.74 (.94)	1.01	−.10	.96 (1.05)	.75	−.71	.68 (.94)	1.22	.37	1.98 (.99)	−.56	−.81	.24 (.64)	2.91	8.13
CES-D 4*	1.05 (1.05)	.55	−.96	1.27 (1.14)	.25	−1.38	.88 (1.05)	.87	−.57	2.25 (.92)	−1.10	.36	.73 (1.19)	1.19	−.38
CES-D 5	1.15 (.92)	.37	−.71	1.01 (.95)	.57	−.66	1.17 (1.05)	.40	−1.06	2.00 (1.03)	−.61	−.85	.41 (.73)	1.83	2.82
CES-D 6	.79 (.95)	.95	−.20	1.22 (1.11)	.37	−1.21	.77 (.97)	1.02	−.11	2.40 (.82)	−1.15	.29	.44 (.72)	1.73	2.59
CES-D 7	1.08 (.94)	.50	−.68	1.22 (1.10)	.35	−1.20	1.55 (1.08)	−.01	−1.28	2.05 (.99)	−.55	−.96	.59 (.92)	1.51	1.18
CES-D 8*	1.18 (.94)	.37	−.75	1.60 (1.11)	−.15	−1.31	.92 (.97)	.70	−.62	2.24 (.79)	−.76	−.03	.87 (.91)	−0.91	−.81
CES-D 9	.40 (.74)	1.90	2.93	.85 (1.06)	.89	−.59	.38 (.76)	2.11	3.71	1.94 (1.02)	−.50	−.94	.19 (.54)	3.28	11.15
CES-D 10	.60 (.83)	1.28	.81	.85 (1.03)	.90	−.46	.64 (.90)	1.24	.52	1.60 (1.10)	−.14	−1.29	.26 (.61)	2.65	7.06
CES-D 11	1.14 (1.00)	.45	−.88	1.37 (1.11)	.18	−1.31	2.00 (1.11)	−.63	−1.05	2.10 (1.05)	−.77	−.73	.65 (.89)	1.23	.56
CES-D 12*	.97 (.89)	.54	−.60	1.40 (1.02)	.10	−1.09	.81 (.92)	.86	−.27	2.29 (.75)	−.88	.46	.60 (.95)	−1.47	.95
CES-D 13	.78 (.85)	.91	.14	.93 (.94)	.76	−.32	.85 (.98)	.83	−.51	1.38 (1.03)	.12	−1.11	.49 (.82)	1.62	1.66
CES-D 14	1.01 (1.00)	.62	−.75	1.36 (1.15)	.15	−1.42	.72 (.98)	1.09	−.05	2.46 (.76)	−1.36	1.40	.37 (.74)	2.09	3.69
CES-D 15	.47 (.71)	1.49	1.80	.58 (.85)	1.34	.86	.34 (.69)	2.24	4.86	1.20 (1.10)	.32	−1.25	.19 (.56)	3.40	11.98
CES-D 16*	1.27 (1.07)	.33	−1.14	1.40 (1.02)	.13	−1.08	.71 (.90)	1.03	.01	2.39 (.62)	−.51	−.61	.53 (.97)	−1.67	1.39
CES-D 17	.53 (.83)	1.45	1.14	.74 (.99)	1.09	−.06	.49 (.86)	1.68	1.75	1.40 (1.14)	.14	−1.39	.16 (.49)	3.52	12.98
CES-D 18	.96 (.90)	.67	−.35	1.26 (1.07)	.39	−1.08	.82 (.93)	.91	−.13	2.26 (.81)	−.66	−.72	.38 (.66)	1.83	3.09
CES-D 19	.58 (.83)	1.37	1.06	.73 (.99)	1.14	.07	.28 (.63)	2.58	6.80	1.86 (1.08)	−.48	−1.05	.14 (.47)	3.76	15.50
CES-D 20	.91 (.92)	.76	−.30	1.14 (1.01)	.42	−.96	1.04 (.99)	.58	−.75	2.12 (.91)	−.63	−.65	.52 (.78)	1.51	1.69

Notes: S = Skew (Standard Error); K = Kurtosis (Standard Error); CES-D = Center for Epidemiological Studies Depression scale; NHANES = National Health and Nutrition Examination Survey;

* = Reverse score.

#### Testing and modifying previous factor solutions

A series of CFAs was conducted to replicate and test selected factor structures published in previous studies (Table 1) and to extend these previous models by excluding potentially problematic items as suggested by previous research. Specifically, there appears to be consensus throughout the literature that items 15 (i.e., “People were unfriendly”) and 19 (i.e., “I felt that people disliked me”) may warrant removal as they reflect interpersonal difficulties, a dimension not consistent with contemporary diagnostic criteria for depression [1], [4], [11], [14], [21]. Similarly, item 17 (i.e., “I had crying spells”) may warrant removal as it produces robust sex differences in endorsement [25], [28], [29]. Accordingly, previously demonstrated factor structures were tested with and without items 15, 17, and 19. Several previous analyses have also suggested that 2-item factors within the CES-D (Table 1) are inherently unstable [15], [39]. Given the challenges associated with 2-item factors, models including a 2-item factor (e.g., [21], [40]) were tested with and without the 2-item factor utilizing the same procedures (i.e., testing with and without items 15, 17, and 19).

CFAs were conducted separately in each sample to determine whether the structure of the CES-D is generalizable and stable across different applications. The size of the clinical sample was not optimal for CFAs but research supports the applicability of CFAs in samples of as low as 51 participants [41]; moreover, the reliability of the factors and the strength of the communalities between the items facilitate the use of CFAs in this sample. The CFAs were performed with AMOS 18 and data from each of the five samples were inputted in a maximum likelihood estimation procedure. Bollen-Stine bootstrap chi-square and computed bootstrapped parameter estimates with estimates from a maximum-likelihood procedure [45], [46] were also conducted because the data did not exhibit multivariate normality; however, results were comparable to the maximum-likelihood procedure and are excluded for brevity. Each model was evaluated using the following fit indices with 90% confidence intervals (when applicable): 1) chi-square (values should not be significant); 2) chi-square/df ratio (values should be less than 2.0); 3) Comparative Fit Index (CFI; values must be greater than.90, and ideal fits approach or are greater than.95); 4) the Standardized Root Mean Square Residual (SRMR; values must be less than.10 and ideal fits approach or are less than.05); 5) Root Mean Square Error of Approximation (RMSEA; values must be less than.08 and ideal fits approach or are less than.05, with 90% confidence interval values below.10); and 6) Expected Cross-Validation Index (ECVI; when comparing these scores across different models, lower values indicate a closer fit [42], [43]. Evaluations emphasized the latter four fit indices (i.e., CFI, SRMR, RMSEA, and ECVI) [44]. Given the large number of models that were tested, only fit indices for solutions where the CFI exceeded.92 in at least three of the five samples were included for presentation.

## Results

### Internal Consistency

Internal consistency was acceptable for the current undergraduate (Cronbach’s α = .91), community (Cronbach’s α = .94), rehabilitation (Cronbach’s α = .92), clinical (Cronbach’s α = .85), and NHANES (Cronbach’s α = .85) samples. The average inter-item Pearson correlation with the reverse-scored items (i.e., positive affect/anhedonia) was .34 for the undergraduate sample, .43 for the community sample, .38 for the rehabilitation sample, .23 for the clinical sample, and .26 for the NHANES sample. The average inter-item Pearson correlation without the reverse-scored items (i.e., positive affect/anhedonia) was .37 for the undergraduate sample, .44 for the community sample, .40 for the rehabilitation sample, .25 for the clinical sample, and .33 for the NHANES sample. In all cases the average inter-item correlation was relatively low, indicating diversity among the items and supporting notions of more than one latent construct. The lowest inter-item correlation was for the clinical sample and suggests that there may be substantial variation among clinical presentations of these symptoms for persons with a history of depression. Such variation is implicitly supported by DSM-IV-TR diagnostic criteria that allow for high levels of negative affect or high levels of anhedonia to qualify as hallmark criteria for major depressive disorder (i.e., “(1) depressed mood or (2) loss of interest or pleasure”; page 356 [1]).

### Sex Differences on CES-D Items

**Figure 1:**
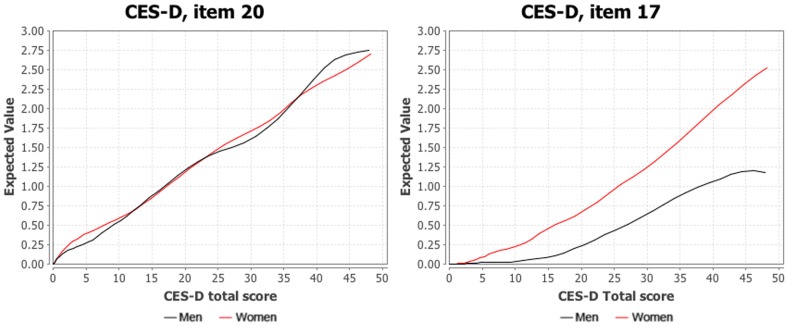
Item characteristic curves.

Across all samples, persons with missing data (i.e., fewer than 1%) were excluded from the analyses. The t-tests comparing men and women’s responses from all samples combined suggested that women reported statistically significantly higher scores (p<.05) on most CES-D items (i.e., 1, 2, 3, 5, 6, 9, 10, 11, 12, 14, 15, 16, 17, 18, 19, 20); however, the effect sizes (i.e., using percentage of variance accounted for “*r* ^2^”) were negligible (i.e., *r* ^2^<.01) for most, but not all items (i.e., items 3, 5, 6, 20, *r* ^2^ = .02; item 14, *r* ^2^ = .03; item 18, *r* ^2^ = .04; item 17, *r* ^2^ = .07). Item 17 (i.e., “I had crying spells”) was the only item with item characteristic curves that differed markedly between men and women, suggesting it has significant differential item functioning. An item with nil or negligible differential item functioning (i.e., item 20) is presented in Figure 1 (i.e., Item characteristic curves) alongside item 17 for illustrative purposes. The item characteristic curves demonstrate that men and women respond similarly to item 17 when depression levels are low or slightly above average (−2.5 SD to +0.5 SD), with both sexes choosing 0 (rarely or none of the time); however, as depression levels increase, women are more likely to choose a higher response option compared to men. Indeed, even the most depressed men are most likely to choose 1 (some or a little of the time), while the most depressed women are more likely to choose 2 (occasionally or a moderate amount of the time) or 3 (most or all of the time). The item characteristic curve plots for all items are not displayed for brevity, but are available from the authors upon request.

### Structural Analyses: CFA Results

**Figure 2:**
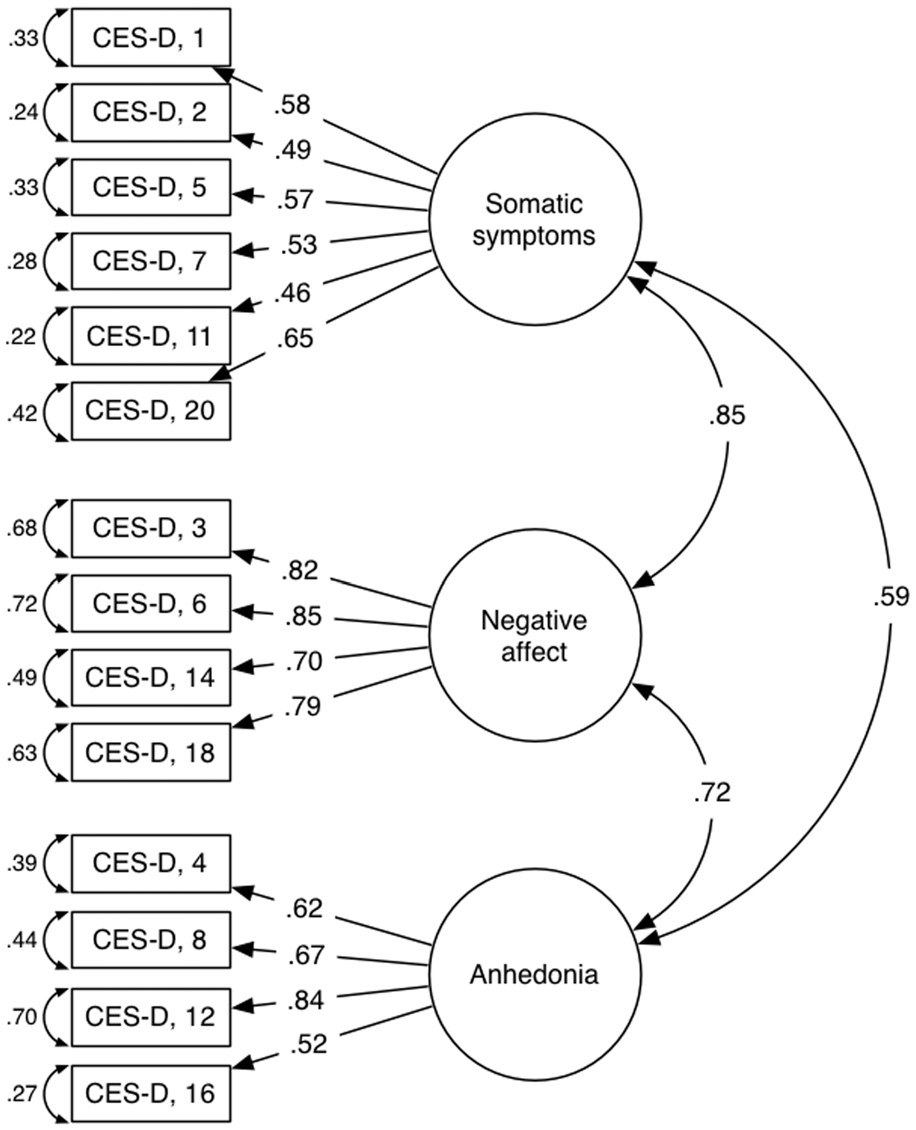
Path Diagram for the CES-D new factor solution.

The fit indices for each of the previously reported models – as evaluated with data from each sample – are presented in Table 3 (where the model CFI exceeded.92 in at least three out of the five samples). The results were interpreted to suggest that five models might have the factorial validity to provide utility in divergent populations, as many of the fit indices met acceptable standards across the different samples. However, all of these models included item 17 and/or failed to include items that assess positive affect, which is inconsistent with current theory and diagnostic approaches concerning depression [1]. Of all the newly derived models (i.e., with items 15, 17, and 19 removed and without 2-item factors [if relevant]), only one exhibited acceptable fit indices within each sample, included positive affect items, and did not include item 17. The model with the best fit indices was a revision of the one proposed by Radloff [47], which also excluded items 9, 10, and 13. Relevant fit indices and inter-factor correlations for this newly derived model are reported in Table 4. The original model proposed by Radloff [47] included four factors: depressed affect (items 3, 6, 14, 17, 18), anhedonia (items 4, 8, 12, 16), somatic complaints (items 1, 2, 5, 7, 11, 20), and interpersonal concerns (items 15, 19). Eliminating item 17 and the two interpersonal items results in an easily interpretable 3-factor structure (Tables 5 and 6; Figure 2– Path Diagram for the CES-D new factor solution) that includes factors of negative affect (items 3, 6, 14, 18), anhedonia (items 4, 8, 12, 16), and somatic complaints (items 1, 2, 5, 7, 11, 20), which is compatible with current DSM-IV-TR conceptualization of depression [1]. The internal consistencies (determined using Cronbach’s alpha) for the total score of the newly derived factor structure (undergraduate α = .87; community α = .92; rehabilitation α = .90; clinical α = .80; NHANES α = .83), the negative affect subscale (undergraduate α = .87; community α = .90; rehabilitation α = .89; clinical α = .82; NHANES α = .74), the anhedonia subscale (undergraduate α = .75; community α = .86; rehabilitation α = .79; clinical α = .81; NHANES α = .73), and the somatic subscale (undergraduate α = .72; community α = .80; rehabilitation α = .78; clinical α = .51; NHANES α = .81) were all acceptable with the exception of the somatic subscale in the clinical sample (i.e., α = .51). The correlation between the total score of the original CES-D and the total score of the current variant, as well as the correlations between their respective subscale scores, were all very high (Table 7).

**Table 3:** CFA fit indices of prior models using current samples and sorted by publication date.

Reference	Factors (Items)	Sample	X^2^	df	X^2^/df	CFI	SRMR	RMSEA	RMSEA 90% CI	ECVI	ECVI 90% CI
Radloff, 1977 Model B [2]	4 (20)†	Undergraduate	741.50	164	4.52	.92	.04	.06	.06;.07	.88	.80;.97
		Community	335.38	164	2.05	.94	.04	.06	.05;.07	1.69	1.50; 1.91
		Rehabilitation	501.36	164	3.06	.93	.05	.06	.06;.07	1.13	1.02; 1.28
		Clinical	218.02	164	1.33	.89	.08	.06	.04;.08	3.74	3.32; 4.25
		NHANES	1209.22	164	7.37	.94	.04	.05	.05;.05	.46	.42;.50
Shrout, 1989 Model A [58]	1 (5)‡ †	Undergraduate	4.76	5	.95	1.00	.01	<.01	<.01;.04	.03	.03;.04
		Community	4.45	5	.89	1.00	.02	<.01	<.01;.08	.10	.10;.13
		Rehabilitation	24.58	5	4.92	.97	.04	.09	.05;.12	.09	.06;.12
		Clinical	4.54	5	.91	1.00	.05	<.01	.00;.15	.30	.30;.41
		NHANES	102.06	5	20.41	.97	.03	.08	.06;.10	.04	.03;.06
Burnam, 1988 [66]; Tuunainen, 2001 [67]	1 (6)†	Undergraduate	74.82	9	8.31	.95	.04	.10	.08;.12	.12	.09;.16
		Community	18.95	9	2.11	.99	.02	.05	.02;.09	.11	.09;.15
		Rehabilitation	22.95	9	2.55	.99	.02	.06	.03;.08	.09	.07;.13
		Clinical	8.73	9	.97	1.00	.05	.00	.00;.12	.39	.40;.53
		NHANES	60.66	9	6.74	.99	.02	.05	.04;.06	.03	.02;.04
Stansbury, 2006 [11]	3 (16)‡ †	Undergraduate	557.32	101	5.52	.92	.04	.07	.06;.08	.66	.59;.75
		Community	216.94	101	2.15	.94	.04	.07	.06;.08	1.13	.98; 1.32
		Rehabilitation	351.30	101	3.48	.94	.05	.07	.06;.08	.81	.71;.93
		Clinical	119.82	101	1.19	.94	.07	.05	.00;.08	2.29	2.06; 2.50
		NHANES	833.84	101	8.26	.95	.03	.05	.05;.05	.32	.29;.36
Lee SW, 2008 Model B [13]	3 (16)‡ †	Undergraduate	562.94	101	5.57	.92	.04	.07	.06;.08	.67	.59;.75
		Community	261.13	101	2.59	.92	.05	.08	.07;.09	1.31	1.14; 1.51
		Rehabilitation	402.09	101	3.98	.92	.05	.08	.07;.08	.91	.80; 1.03
		Clinical	122.00	101	1.21	.94	.07	.05	.00;.08	2.31	2.06; 2.70
		NHANES	990.73	101	9.81	.94	.04	.06	.05;.06	.38	.34;.41

Notes: For clarity, the first author’s name and the year are provided to help identify models; Underlined indices met stated minimum fit criteria;

‡ = Model did not include the positive affect items;

† = Model includes item 17; NHANES = National Health and Nutrition Examination Survey; X^2^ =  Chi-Square; X^2^/df = Chi-Square/df ratio; CFI = Comparative Fit Index; SRMR = Standardized Root Mean Square Residual; RMSEA = Root Mean Square Error of Approximation; ECVI = Expected Cross-Validation Index; CI = Confidence Interval.

**Table 4:** Newly derived 3-factor 14-item solution and associated CFA fit indices.

								Inter-factor Correlations		
Sample	X^2^	df	X^2^/df	CFI	SRMR	RMSEA (90% CI)	ECVI (90% CI)	1∶2	2∶3	1∶3
Undergraduate	286.79	74	3.88	.96	.04	.06 (.05;.06)	.37 (.32;.43)	.79	.57	.75
Community	151.35	74	2.05	.96	.04	.06 (.05;.08)	.84 (.72; 1.00)	.83	.66	.74
Rehabilitation	174.64	74	2.36	.97	.04	.05 (.04;.06)	.45 (.39;.54)	.84	.71	.80
Clinical	87.24	74	1.18	.96	.07	.05 (<.01;.08)	1.80 (1.64; 2.13)	.85	.60	.46
NHANES	556.11	74	7.52	.96	.04	.05 (.04;.05)	.22 (.19;.25)	.87	.35	.30

Notes: Underlined indices met stated minimum fit criteria; NHANES = National Health and Nutrition Examination Survey; X^2^ =  Chi-Square; X^2^/df = Chi-Square/df ratio; CFI = Comparative Fit Index; SRMR = Standardized Root Mean Square Residual; RMSEA = Root Mean Square Error of Approximation; ECVI = Expected Cross-Validation Index; CI = Confidence Interval.

**Table 5:** The 14 items from the original CES-D included in the new solution and their assigned factors.

CES-D Original items	New three factors
1. I was bothered by things that usually don’t bother me.	3
2. I did not feel like eating; my appetite was poor.	3
3. I felt that I could not shake off the blues, even with the help from family or friends.	1
4. I felt that I was just as good as other people.	2
5. I had trouble keeping my mind on what I was doing.	3
6. I felt depressed.	1
7. I felt that everything I did was an effort.	3
8. I felt hopeful about the future.	2
9. I thought my life had been a failure.	-
10. I felt fearful.	-
11. My sleep was restless.	3
12. I was happy.	2
13. I talked less than usual.	-
14. I felt lonely.	1
15. People were unfriendly.	-
16. I enjoyed life.	2
17. I had crying spells.	-
18. I felt sad.	1
19. I felt that people disliked me.	-
20. I could not get “going”.	3

Notes: CES-D – Center for Epidemiological Studies Depression scale; Factor 1 =  negative affect; Factor 2 =  anhedonia; Factor 3 =  somatic symptoms.

**Table 6:** Loading weights and residuals for the CES-D new factor solution.

		Undergraduate Sample	Community Sample	Rehabilitation Sample	Clinical Sample	NHANES Sample
	Item Number	Weight (Residual)	Weight (Residual)	Weight (Residual)	Weight (Residual)	Weight (Residual)
Somatic Symptoms	1	.58 (.33)	.59 (.35)	.72 (.52)	.56 (.31)	.59 (.35)
	2	.49 (.24)	.57 (.33)	.61 (.37)	.09 (.01)	.51 (.26)
	5	.57 (.33)	.63 (.39)	.65 (.43)	.56 (.31)	.64 (.41)
	7	.53 (.28)	.73 (.53)	.56 (.32)	.50 (.25)	.59 (.36)
	11	.46 (.22)	.59 (.35)	.38 (.14)	.11 (.01)	.59 (.36)
	20	.65 (.42)	.73 (.53)	.70 (.48)	.58 (.33)	.70 (.49)
Negative Affect	3	.82 (.68)	.88 (.77)	.83 (.69)	.68 (.46)	.74 (.55)
	6	.85 (.72)	.88 (.77)	.88 (.77)	.89 (.80)	.84 (.71)
	14	.70 (.49)	.70 (.49)	.73 (.54)	.61 (.38)	.74 (.55)
	18	.79 (.63)	.86 (.74)	.85 (.72)	.79 (.36)	.81 (.66)
Anhedonia	4	.62 (.39)	.62 (.39)	.54 (.29)	.62 (.39)	.47 (.22)
	8	.67 (.44)	.68 (.46)	.58 (.33)	.59 (.35)	.57 (.33)
	12	.84 (.70)	.91 (.82)	.85 (.72)	.82 (.67)	.80 (.64)
	16	.52 (.27)	.88 (.78)	.82 (.67)	.87 (.75)	.77 (.60)

**Table 7:** Inter-subscale correlations for the new CES-D factor solution.

	CES-D Total	CES-DR Total	CES-DR Negative Affect	CES-DR Anhedonia
Undergraduate Sample				
CES-DR Total	.98	–		
CES-DR Negative Affect	.90	.89	–	
CES-DR Anhedonia	.72	.76	.55	–
CES-DR Somatic	.84	.85	.68	.41
Community Sample				
CES-DR Total	.98	–		
CES-DR Negative Affect	.91	.91	–	
CES-DR Anhedonia	.79	.82	.65	–
CES-DR Somatic	.87	.87	.71	.53
Rehabilitation Sample				
CES-DR Total	.99	–		
CES-DR Negative Affect	.91	.89	–	
CES-DR Anhedonia	.76	.80	.64	–
CES-DR Somatic	.86	.88	.68	.51
Clinical Sample				
CES-DR Total	.95	–		
CES-DR Negative Affect	.88	.87	–	
CES-DR Anhedonia	.60	.70	.54	–
CES-DR Somatic	.75	.80	.55	.24†
NHANES				
CES-DR Total	.98	–		
CES-DR Negative Affect	.83	.79	–	
CES-DR Anhedonia	.59	.67	.23	–
CES-DR Somatic	.81	.80	.68	.16

Notes: CES-D = Center for Epidemiological Studies Depression scale; CES-DR = new solution of the Center for Epidemiological Studies Depression scale; NHANES = National Health and Nutrition Examination Survey;

† p>.01, all other correlations p<.01.

## Discussion

Despite the popularity of the CES-D, there has been considerable debate regarding the optimal factor structure and item content for the measure (see Table 1). The current study sought to summarize and address these issues by assessing the differential validity of the CES-D and comparing the previously proposed factor solutions for the CES-D to a novel, theoretically-driven model. The results support a 14-item, 3-factor model that is relatively more congruent with current diagnostic criteria for depression [1].

Previous research has highlighted that item 17 (i.e., “I had crying spells”) of the CES-D may lead to inflated scores for women [19], [20], [25], [28], [29]. As expected, item 17 exhibited significant differential item functioning, such that even the most depressed men were most likely to choose 1 (some or a little of the time) on the Likert scale for that item, compared to the most depressed women, who were more likely to choose 2 (occasionally or a moderate amount of the time) or 3 (most or all of the time). This finding underscores the importance of removing item 17 from the CES-D and subsequently creating and utilizing new norms for the measure that do not include this item. Continued use of item 17 and the associated norms or cut-offs will lead to notable overestimates of depression in women and underestimates of depression in men. Such misrepresentations owing to sex and cultural biases, rather than true differences in depression, may have significant social and practical healthcare implications. Attempting to control for this sex difference by subtracting a value from women’s scores (e.g., one point off of the total), or by otherwise adjusting norms for each sex would be inappropriate because sex differences on this item are nonlinear (i.e., women score higher compared to men when both are severely depressed). To illustrate, removing one point from women’s scores would substantially and inappropriately lower scores of women who are on the lower spectrum of depression (i.e., because item 17 is less biased on the lower end of the spectrum) and would still overestimate the severity of depression in severely depressed women when compared to men.

Results of the CFAs failed to support CES-D models previously identified by exploratory factor analyses. All models with minimally acceptable fit indices for three out of the five samples included individual items or 2-item factors that previous research suggests should not be included in the CES-D [25], [28], or involved extreme reductions in item content that impede the capacity of the CES-D to assess DSM-IV-TR depressive symptoms [1]. A modified version of the model proposed by Radloff [47] provided a 3-factor (i.e., negative affect, anhedonia, and somatic symptoms), 14-item solution that is consistent with contemporary conceptualization of depression [1] and demonstrated excellent fit within all samples as indicated by all fit indices. The solution also exhibited acceptable internal consistency for all factors within all samples, with the exception of the somatic factor having relatively poor internal consistency within the sample with a history of depression. The differing results for internal consistency suggest that negative affect and anhedonia may be the most characteristic and consistent symptoms of depression, while somatic symptoms may be more variable between individuals with a history of depression. The differences may result from somatic symptoms being endorsed for reasons other than depression, such as chronic pain.

Several theoretical and clinical implications follow the present findings. Researchers and clinicians should not use item 17 of the CES-D (i.e., “I had crying spells”) or be careful of its use and interpretation. As the current results illustrate, a women crying is not necessarily a viable index of her depression severity − perhaps owing to culture norms of emotional expression − and a lack of crying in either sex is not a viable index of an absence of depression. Utilizing item 17 may lead to skewed estimations of depression and invalid cut-offs scores. Nevertheless, crying is a symptom of emotional distress, and researchers should explore the possibility of creating a new item that assesses frequency of crying without a sex bias. For example, perhaps a relative measure of crying (e.g., “I cried much more frequently than I usually do” or “I felt like crying more than usual”) rather than an absolute measure of crying (e.g., “I cried most of the time”) may limit such sex biases. Moreover, the current model is consistent with previous findings suggesting that socially-focused items of the CES-D (i.e., items 15 and 19) should not be included in the measure [4], [11], [14], [21]. Finally, the current results further support depression as a multidimensional disorder consisting of negative affect, anhedonia, and somatic symptoms [48]–[50].

The review of prior studies on the factor structure of the CES-D highlights the divergent results of previous exploratory factor analyses, none of which were strongly supported by CFAs with the present data. Future studies of the CES-D may benefit more from conducting further theory-driven confirmatory analyses rather than exploratory analyses. The majority of previously reported factor solutions suggested by previous exploratory factor analyses exhibited poor fit in the current samples. The best fitting solution was derived from contemporary theoretical research and previously established empirical data and exhibited excellent fit in the variety of samples used. Accordingly, the version of the CES-D presented herein would likely maintain factorial validity across different settings (e.g., clinical, research). Future research on the CES-D would benefit from exploring different forms of validity (e.g., convergent validity, predictive validity) with the item set from the model suggested here. In addition, future research designs should explicitly include comments regarding the influence of sample on factor structure fit indices – a variable that the current results indicates is important.

Several limitations of the current study provide directions for future research. First, the majority of participants in the current samples were not formally evaluated (e.g., with a structured clinical interview) for clinically significant depression and although the diagnostic criteria for depression has changed minimally since data for the NHANES was collected (roughly 37 years ago), potential changes over time with respect to social and cultural attitudes may have resulted in different response rates and patterns than if this data was collected today. Future research should assess the sensitivity and specificity of the proposed item set with participants categorized as meeting or not meeting DSM-IV criteria for Major Depressive Disorder. Second, the inability to clinically classify individuals with or without depression also precluded estimation of appropriate cut-off scores for the CES-D. Future research may benefit from re-examining cut-off scores while removing items identified in the current paper as inappropriate. Such an examination may shed light on discrepancies in recommendations for cut-off scores [51]–[56]. Third, including the reverse-scored items that are straightforwardly worded assessments of positive affect/anhedonia may be creating a psychometric bias as a result of incidental response errors. Such a possibility is relatively less likely than using reverse-worded items, but future research could assess for such a bias by examining the items separately and adding a measure that is not based entirely in self-report for convergent and divergent validity. Fourth, combining all five samples created a large enough sample to produce accurate estimations of differential item functioning; however, the combination of differing samples (e.g., clinical, community) may have introduced unmeasured confounds (e.g., cultural differences in the NHANES but not in the clinical sample) that may impact differential item functioning. Future research should examine differential item functioning on the CES-D in a variety of large, culturally homogeneous samples. Fifth, the current study only provides support for a revised version of the CES-D in a primarily English-speaking sample. Future research should cross-validate this revision using a more culturally diverse sample and test its compatibility with versions of the CES-D in other languages. Sixth, the somatic factor included in the final solution demonstrated adequate fit, but relatively low internal consistency. As such, the somatic items may benefit from further revision as they may currently focus on symptoms that are also characteristic of other disorders (e.g., anxiety disorders) or fail to assess symptoms frequently associated with depression. For example, item 11 (i.e., “My sleep was restless”) is too vague to be specifically related to depression and certainly excludes hypersomnia, waking early, and difficulty falling asleep, which are characteristic of depression [1]. Additional revisions to CES-D content might also consider including items describing cognitive symptoms of depression (e.g., thoughts of worthlessness or suicidal ideation) to further adhere to current diagnostic criteria. It may also be worthwhile for future researchers to consider adopting a differential weighting schema for items in the CES-D, such that items are weighted according to their analytical power. That said, given the increasing availability of alternative screening measures (e.g., PHQ-9 [57]), coupled with the longstanding psychometric difficulties of the scale, it may be time to begin the process of retiring the CES-D in favor of newer measures that are also freely available for use.

The present study addressed pertinent issues associated with CES-D items and precedent factor structures. CFAs performed with several samples (i.e., undergraduate, community, rehabilitation, clinical, and NHANES) were interpreted to suggest a novel best fitting model for the CES-D that is psychometrically and theoretically robust, comprising 3-factors (i.e., negative affect, anhedonia, somatic symptoms) and 14-items relatively more congruent with current diagnostic criteria for depression [1]. The CES-D items may benefit from additional revision; however, this alternative solution offers a valid item set, without biases related to social concerns or sex, for research and clinical applications.
